# Minding the gap in HIV host genetics: opportunities and challenges

**DOI:** 10.1007/s00439-020-02177-9

**Published:** 2020-05-14

**Authors:** Shanelle N. Gingras, David Tang, Jeffrey Tuff, Paul J. McLaren

**Affiliations:** 1 JC Wilt Infectious Diseases Research Centre, National HIV and Retrovirology Lab, National Microbiology Laboratories, Public Health Agency of Canada, Winnipeg, Canada; 2 Department of Medical Microbiology and Infectious Diseases, University of Manitoba, Winnipeg, Canada

## Abstract

Genome-wide association studies (GWAS) have been successful in identifying and confirming novel genetic variants that are associated with diverse HIV phenotypes. However, these studies have predominantly focused on European cohorts. HLA molecules have been consistently associated with HIV outcomes, some of which have been found to be population specific, underscoring the need for diversity in GWAS. Recently, there has been a concerted effort to address this gap that leads to health care (disease prevention, diagnosis, treatment) disparities with marginal improvement. As precision medicine becomes more utilized, non-European individuals will be more and more disadvantaged, as the genetic variants identified in genomic research based on European populations may not accurately reflect that of non-European individuals. Leveraging pre-existing, large, multiethnic cohorts, such as the UK Biobank, 23andMe, and the National Institute of Health’s All of Us Research Program, can contribute in raising genomic research in non-European populations and ultimately lead to better health outcomes.

## Genomic studies in HIV: progress to date

Since the completion of the human genome project, genome-wide association studies (GWAS) have identified thousands of reproducible genetic associations with hundreds of complex human traits (Collins et al. 2004; MacArthur et al. 2018). Although chronic diseases, such as type 2 diabetes, have amassed tremendously large sample sizes resulting in > 100 associated loci (Xue et al. 2018), infectious diseases have only begun to be explored to the same level despite being a major cause of morbidity and mortality globally (Wang et al. 2016). The first GWAS in infectious diseases, performed in people living with HIV (PLWH), identified two independent genetic variants located in human leukocyte antigen (HLA)-B and -C that associated with set point viral load (spVL). This phenotype, defined as mean copies of the HIV RNA genome detected in untreated individuals during the chronic phase of infection (Fellay et al. 2007), correlates both with rate of disease progression to acquired immunodeficiency disease (AIDS) (Mellors et al. 1995) and with transmission potential (Quinn et al. 2000). The observed genetic variants explained 9.6% and 6.5% of the variation seen, respectively, and held promise that natural models of HIV control could be informative for design of novel HIV intervention strategies (Fellay 2009; Fellay et al. 2007). Since that first study, technological advances in genome-wide platforms have improved the ability to detect and confirm genetic variants identified in GWAS, however, the majority of these studies have been conducted in populations of primarily European descent (Table 1) (MacArthur et al. 2018). The major phenotypes explored in HIV GWAS are: susceptibility to infection, spVL, and disease progression, measured as rate of CD4 T-cell decline (Le Clerc et al. 2009; Herbeck et al. 2010; van Manen et al. 2011). However, studies of these critical phenotypes are much more commonly carried out in European cohorts (Table 1). Furthermore, the majority of studies that do focus on a multiethnic cohort have a greater proportion of European individuals included. With a predominant focus on European individuals, genetic variants driving HIV phenotypes may go undetected.

**Table 1:** Genome-wide association studies on human immunodeficiency virus-1 infection

First author	Publication date (yyyy-mm-dd)	Journal	Reported trait	Sample number (ancestry)	Cohort used	References
European Ancestry Cohort						
Fellay	2007-07-19	Science	HIV viral setpoint	486 European	EuroCHAVI	Fellay et al. (2007)
Limou	2000-01-01	J Infect Dis	AIDS progression	1627 European, 86 NR	GRIV	Limou et al. (2009)
Le Clerc	2009-09-15	J Infect Dis	AIDS	2134 European	GRIV	Le Clerc et al. (2009)
Fellay	2009-12-24	PLoS Genet	HIV-1 control	2362 European	EuroCHAVI	Fellay (2009)
Ferreira	2009-12-31	AM J Hum Genet	CD56 positive NK-lymphocyte cell count CD19-positive B-lymphocyte count CD8-positive T-lymphocyte count CD4-positive T-lymphocyte count	2538 European	Siblings from 1089 Australian families	Ferreira et al. (2010)
Shrestha	2010-01-01	AIDS	Carotid atherosclerosis in HIV infection	177 European	FRAM	Shrestha et al. (2010)
Herbeck	2010-02-15	J Infect Dis	HIV-1 progression	156 European	MACS	Herbeck et al. (2010)
Bol	2011-02-25	PLoS One	HIV-1 replication	191 European	Healthy blood donors	Bol et al. (2011)
Troyer	2011-05-01	J Infect Dis	AIDS progression	755 European	GRIV	Troyer et al. (2011)
van Manen	2011-07-21	PLoS One	HIV-1 progression	404 European	ACS	van Manen et al. (2011)
Irvin	2011-12-01	Pharmacogenet Genomics	Fat distribution (HIV)	192 European	FRAM	Irvin et al. (2011)
Lane	2013-02-10	Hum Mol Genet	HIV-1 susceptibility	1195 European	CHAVI014, MHCS	Lane et al. (2013)
McLaren	2013-07-25	PLoS Pathog	HIV-1 susceptibility	13,581 European	ACTG; ALIVE; ACS; ANRS CO18; ANRS PRIMO Cohort; CCR; CHAVI; Danish HIV Cohort Study; GISHEAL; GRIV Cohort; HGDS; Hospital Clinic-IDIBAPS Acute/Recent HIV-1 Infection; Icona Foundation Study; International HIV Controllers Study, The IrsiCaixa Foundation Acute/Recent HIV-1 Infection Cohort; Modena Cohort; MACS; MHCS; Pumwani Sex Workers Cohort; SFCCC; The GPC Cohort/ Sanger Institute in Oxford, UK, and in Uganda; SHCS; University Medical Center Utrecht Dutch Controls Cohort; NHS; WTCCC3; Western Australian HIV Cohort Study	McLaren et al. (2013)
McLaren	2015-11-09	Proc Natl Acad Sci U S A	Setpoint viral load in HIV-1 infection	6315 European	EuroCHAVI, ALIVE, DCG, HGDS, MHCS, SFCC, GRIV, PRIMO, MACS, ACTG, IHCS, ACS, UHS	McLaren et al. (2015)
Ulveling	2016-07-29	Hepatology	Liver fibrosis severity in HIV/hepatitis C co-infection	289 European	French National Agency for Research on AIDS and Viral Hepatitis CO13 HEPAVIH	Ulveling et al. (2016)
African Ancestry Cohorts						
Pelak	2010-04-15	J Infect Dis	HIV-1 viral setpoint	515 African American or Afro-Caribbean	NHS; MACS	Pelak et al. (2010)
Petrovski	2011-02-20	AIDS	HIV-1 susceptibility	1379 Sub-Saharan African	Recruited from the Center for HIV/AIDS Vaccine Immunology Clinical Core in Malawi	Petrovski et al. (2011)
Lingappa	2011-12-12	PLoS One	HIV-1 susceptibility	798 Sub-Saharan African, African unspecified	The Partners in Prevention HSV/HIV Transmission Study; The Couples Observational Study	Lingappa et al. (2011)
Carr	2017-01-05	J Antimicrob Chemother	Nevirapine-induced hypersensitivity in HIV (drug-induced liver injury)	203 Sub-Saharan African	Recruited from the Queen Elizabeth Central Hospital, Malawi	Carr et al. (2017)
			Nevirapine-induced hypersensitivity in HIV	333 Sub-Saharan African		
			Nevirapine-induced hypersensitivity in HIV (rash)	398 Sub-Saharan African		
			Nevirapine-induced hypersensitivity in HIV (hypersensitivity syndrome)	205 Sub-Saharan African		
			Nevirapine-induced hypersensitivity in HIV (Stevens-Johnson syndrome or toxic epidermal necrosis)	233 Sub-Saharan African		
Asian Ancestry Cohorts						
Uttayamakul	2015-06-15	AIDS Res Hum Retroviruses	Response to stavudine in HIV (lipoatrophy)	141 Southeast Asian	Bamrasnaradura Infectious Diseases Institute, Nonthaburi, Thailand	Uttayamakul et al. (2015)
Wei	2015-06-17	Sci Rep	HIV-1 viral setpoint	538 East Asian	Chinese Medical University: HAN, YUN, XIN	Wei et al. (2015)
Multiethnic cohorts						
Pereyra	2010-11-04	Science	HIV-1 control	1712 European; 1233 African American or Afro-Caribbean; 677 Hispanic or Latin American	International HIV Controllers Study, ACTG	Pereyra et al. (2010)
Moore	2015-01-09	Open Forum Infect Dis	Neutrophil count in HIV infection; HDL cholesterol in HI -infection; total bilirubin levels in HIV-1 infection	2547 European, Hispanic or Latin American, African unspecified	ACTG	Moore et al. (2015)
Lehmann	2015-02-01	Pharmacogenet Genomics	Response to abacavir-containing treatment in HIV-1 infection (virologic failure)	346 European; 265 African unspecified; 175 Hispanic or Latin American	ACTG	Lehmann et al. (2015)
Lehmann	2015-02-01	Pharmacogenet Genomics	Response to efavirenz-containing treatment in HIV-1 infection (virologic failure)	737 European; 545 African unspecified; 314 Hispanic or Latin American	ACTG	Lehmann et al. (2015)
Johnson	2015-03-18	PLoS One	HIV-1 susceptibility	2004 African American or Afro-Caribbean; 1132 European	UHS	Johnson et al. (2015)
Wanga	2015-09-01	Pharmacogenet Genomics	Bilirubin measurement, response to tenofovir, HIV infection, creatinine clearance	243 European, 149 African American, 100 Hispanic American, 9 UA	ACTG	Wanga et al. (2015)
Jia	2017-04-26	Am J Med Genet B Neuropsychiatr Genet	Neurocognitive impairment in HIV-1 infection (continuous)	490 African American or Afro-Caribbean; 443 European; 99 Hispanic or Latin American; 17 NR	CHARTER	Jia et al. (2017)
Jia	2017-04-26	Am J Med Genet B Neuropsychiatr Genet	Neurocognitive impairment in HIV-1 infection (dichotomous)	490 African American or Afro-Caribbean; 443 European; 99 Hispanic or Latin American; 17 NR	CHARTER	Jia et al. (2017)
Ekenberg	2019-06-13	J Infect Dis	Pre-treatment viral load in HIV-1 infection	572 African unspecified; 1398 European; 418 Hispanic or Latin American; 13 Asian unspecified; 39 NR	START	Ekenberg et al. (2019)

GWAS were compiled through the NHGRI-EBI GWAS Catalog using the search terms “HIV-1” and “AIDS”. Cohorts used have been abbreviated. All abbreviations are listed below in alphabetical order. ‘NR’ is not reported ancestry, ‘UA’ is unknown ancestry (MacArthur et al. 2018)

*ACS* Amsterdam Cohort Studies on HIV Infection and AIDS, *ACTG* AIDS Clinical Trials Group, *ALIVE* AIDS Linked to the IntroVenous Experience, *CCR* Center for Cancer Research, National Cancer Institute, *CHARTER* CNS HIV Antiretroviral Therapy Effects Research, *CHAVI* Center for HIV/AIDS Vaccine Immunology, *EuroCHAVI Consortium* Swiss HIV Cohort Study; Icona Foundation Study; IrsiCaixa Foundation; San Raffaele del Monte Tabor Foundation; Danish Cohort; Royal Perth Hospital: Guy's, King's, and St. Thomas' Hospitals; Modena Cohort; Hospital Clinic-IDIBAPS cohort; Multicenter AIDS Cohort Study, *FRAM* Fat Redistribution and Metabolic Change in HIV Infection, *GISHEAL* The Genetic and Immunological Studies of European and African HIV-1 Long Term Non-Progressors, *HGDS* Hemophilia Growth and Development, *MACS* Multicenter AIDS Cohort Study, *MHCS* Multicenter Hemophilia Cohort Study, *NHS* The US Military HIV Natural History Study, *SFCCC* San Francisco City Clinic Cohort, *SHCS* Swiss HIV Cohort Study, *START* Strategic Timing of AntiRetroviral Treatment, *UHS* Urban Health Study, *WTCCC3* The Wellcome Trust Case Control Consortium study of the genetics of host control of HIV-1 infection in the Gambia

The largest GWAS done in HIV has been conducted by the International Collaboration for the Genomics of HIV, which has assessed the host genetic contribution to HIV acquisition and spVL (McLaren et al. 2013, 2015). For HIV acquisition, although no genome-wide significant variants were identified by comparing > 6000 HIV-infected individuals to > 7000 general population controls, a subsequent analysis did identify significant heritability due to common genetic variants, suggesting larger more diverse samples may uncover novel associations (McLaren et al. 2013; Power et al. 2017). Aggregating data across studies can increase the power to detect associations by addressing the issue of sample size, however, there are many factors to consider for aggregation and subsequent meta-analysis. Data access and exchange can be a difficult balance between protecting the study participants and providing enough information to be useful to the wider scientific community without data breaches, reidentification, or misuse (Zook et al. 2017). Ultimately sharing data positively outweighs the challenges, however, with the stigmatizing nature of an HIV diagnosis, it is imperative that researchers take the proper precautions to protect individuals’ privacy. To address these concerns, studies now offer very broad consent forms to enable the future use and exchange of information and there are protocols that must be followed for data exchange and storage to limit the possibility of data breaches (Zook et al. 2017). Meta-analysis historically has required a homogenous population of study, which is problematic for individuals of African ethnicity as diversity is not only higher than the European population, but they are also less represented in genomic research, leading to smaller potential for data aggregation of a homogenous population (Popejoy and Fullerton 2016; Russo 2007). Statistical methods have been developed that not only account for the differences observed between populations (allelic heterogeneity, linkage disequilibrium (LD) patterns, effect sizes), but also takes into account different genotyping platforms (Wang et al. 2012). It is also important to consider overlap of individuals between studies and cohorts when aggregating data together. As we can see in Table 1, GWAS for HIV reuse multiple cohorts and subsets of cohorts for various studies. Aggregation of these data would require accounting for each individual overlap to ensure no bias is introduced. Aggregating the large amount of data available in HIV genomics would be no easy feat, but looking to current databases that have freely available genome and exome data (Genome Aggregation Database (gnomAD), The 1000 Genomes Project Consortium) can serve as an example for data aggregation (Auton et al. 2015; Karczewski et al. 2019). Exome and genome sequencing projects should be undertaken to address the role of rare variation that is not well captured by GWAS, and with a growing number of sequencing projects, it would serve the scientific community well to aggregate these data for future large-scale use.

For spVL, in addition to reaffirming the protective role of HLA-B*57:01, B*27:05, B*14:02 among other alleles, analysis mapping individual amino acid residues in the HLA-A and B binding grooves showed that strong effects on spVL were mediated by variable residues (McLaren et al. 2015). As with this largest study, the majority of GWAS of HIV spVL and disease progression have underscored the importance of HLA class I genes in controlling HIV replication and limiting disease progression. HLA class I molecules are responsible for peptide binding specificity and variation within this region has important implications in disease pathogenesis (Pelak et al. 2010; Peprah et al. 2015; Pereyra et al. 2010; Ramsuran et al. 2018). Multiple variants within class I HLA molecules have been found to reduce spVL and be protective for disease progression in HIV, including the population-specific HLA-B*57:01 for Europeans and HLA-B*57:03 for Africans (Fellay 2009; Fellay et al. 2007; McLaren et al. 2013; Pelak et al. 2010; Pereyra et al. 2010). The mechanism of protection attributed to HLA-B alleles has been proposed to be through a more diverse recognition of a broader epitope repertoire and through recognition of more conserved HIV peptides (Arora et al. 2019; Gaiha et al. 2019). However, it has also been proposed that class I HLA alleles may exert non-peptide dependent protective effects. Recently, Ramsuran et al. investigated the impact of HLA-A on control of HIV and its interaction with other HLA molecules (HLA-E and -B) in an ethnically diverse cohort (2018). Similar to HLA-C, HLA-A is differentially expressed based on allotype specificity (Apps et al. 2013; Ramsuran et al. 2018). However, in contrast to HLA-C, the surface expression of HLA-A is 13- to 18-fold higher and is twofold more polymorphic (Ramsuran et al. 2018). The authors observed that HLA-A expression levels positively associated with a higher VL across populations in spite of varying allelic frequencies, an association that was confirmed in a cohort of individuals with known seroconversion dates (Ramsuran et al. 2018). Furthermore, increased HLA-A expression level was strongly associated with elevated mean VL, increased odds of being an HIV non-controller versus controller, and decreased CD4 + T-cell counts (Ramsuran et al. 2018).

The mechanism underlying the impact of HLA-A on HIV progression was further investigated in the context of its relationship to HLA-E. HLA-E is a ligand for the CD94/NKG2A receptor found on both natural killer (NK) cells and T cells. When stably bound to a signal peptide produced by the leader sequences of HLA-A, -B, and -C, HLA-E strongly inhibits NK cells activity (Lee et al. 1998; Ramsuran et al. 2018). Interestingly, highly expressed HLA-A alleles significantly correlated with higher expression of HLA-E on the cell surface, independent of the known protective HLA-B alleles, and reduced the control of HIV via NKG2A-mediated NK inhibition by interfering with the clearance of HIV-infected cells (Ramsuran et al. 2018). HIV favors the increased expression of HLA-E to decrease the risk of NK cell recognition, yet this immune evasion strategy heightens the risk of restricted CD8 + T-cell recognition (Hansen et al. 2016). Recently, researchers have been able to exploit this mechanism by eliciting unconventional CD8 + T-cell responses to MHC-E peptide presentation in rhesus macaques (RM) using a rhesus cytomegalovirus (RhCMV) SIVgag vector-based vaccine (Hansen et al. 2013, 2016; Walters et al. 2018). This vaccine broadly enables CD8 + T cells to recognize MHC-E-restricted presentation of highly variable peptides and can be targeted for a pathogen’s specific vulnerability through deletions in the RhCMV genome, in the case of HIV, the upregulation of MHC-E on the cell surface (Früh and Picker 2017; Hansen et al. 2013, 2016; Walters et al. 2018). With more than 50% of the vaccinated RM demonstrating protection to a highly pathogenic SIV strain and infected RM being indiscernible from vaccinated unchallenged RM leading up to 18 months post-infection, targeting of HLA-E upregulation during HIV infection is an attractive strategy moving forward (Früh and Picker 2017; Hansen et al. 2013, 2016; Walters et al. 2018). These data demonstrate the myriad of ways in which HLA may contribute to control and clearance of infection.

Multiple independent genetic associations have also been reported in the CCR5 gene region. The strongest of which, CCR5∆32 confers protection against infection in the homozygous state and slows disease progression in heterozygotes (Dean et al. 1996). Recently, Kulkarni et al. demonstrated that an additional polymorphism in the same region, rs1015164 which also impacts spVL (McLaren et al. 2015), associated with expression of an antisense long non-coding RNA that overlaps CCR5, dubbed CCR5-AS (2019). Expression level of CCR5-AS positively associated with expression of CCR5 and, when depleted, led to a reduction in HIV infection in vitro highlighting an alternative mechanism for CCRR5-related HIV control (Kulkarni et al. 2019).

Host control of HIV has been shown to differ across ethnicities likely due in part to population-specific variants associated with susceptibility to infection and disease progression (Peprah et al. 2015; Pereyra et al. 2010). Frequency and effect size of risk alleles can vary substantially across populations, making it critical to identify associated genetic variation in non-European populations, particularly in Africa where HIV is found in the highest proportion worldwide (Peprah et al. 2015; UNAIDS 2019). Closing this gap is not only important to improve health disparities, but can also improve genetic discovery by uncovering risk alleles not found in European populations. Recently, efforts have been made to improve ethnic diversity in HIV genomic studies. Ekenberg et al. assessed genetic regulators of VL on a subset of the Strategic Timing of Antiretroviral Treatment (START) cohort, that enrolled ART-naïve, ethnically diverse (35 countries from 5 continents), HIV-positive individuals (Ekenberg et al. 2019). Five SNPs met genome-wide significance, all of which were found in the MHC class I region (Ekenberg et al. 2019). The most significant SNP, located upstream of HLA-B, was identified to tag HLA-B*5701 (Ekenberg et al. 2019). Importantly, the observed variant exhibited high positive predictive values (PPV) in White, Asian, and Hispanic populations, however, in the Black participants PPV was low (Ekenberg et al. 2019). Two more population-specific variants were also confirmed in this study, HLA-B*5701 and HLA-B*5703, with higher frequencies found in European and African populations, respectively (Ekenberg et al. 2019). Two SNPS that have previously been reported to control HIV infection, one located in the HLA complex P5 gene and another in close proximity to the MICA gene, were found to be in moderate LD with the top associated SNP (Ekenberg et al. 2019). The SNP found close to the MICA gene was also found to be statistically significant across all four ethnicities (Ekenberg et al. 2019). These data demonstrate that although there are variants that consistently associate with phenotypes across populations, there are variants that are specific to a population and unique SNPs can tag HLA alleles distinct to a population due to underlying variations in LD structure, reflecting out of Africa migration patterns and the shortening of African LD blocks through increased generational decay (Charles et al. 2014; Ekenberg et al. 2019).

## Opportunities for novel discovery using large, multiethnic data sets

Currently, there are large multiethnic genetic studies that are ongoing to address the disparity in genomic medicine across ancestry groups. One such study, The UK Biobank, consists of 500,000 UK participants enrolled at ages 40–69 from diverse ethnic backgrounds (UK Biobank 2017). All 500,000 participants have been genotyped and electronic health records (general practice, hospital episodes, cancer, death) have been linked to the participants allowing for the joint analysis of genetic, clinical and demographic data on relevant clinical samples (blood, urine, saliva) taken at the time of enrollment for all participants, and at follow-up appointments for a subset of 20,000 individuals (UK Biobank 2017).

Mentzer et al. used the UK Biobank resource to study the host–pathogen–disease relationship using a Multiplex Serology platform optimized for 20 different pathogens, with three disease endpoints of known (human papillomavirus (HPV)-16 and cervical cancer), potential (Epstein–Barr virus (EBV) and cytomegalovirus (CMV) and multiple sclerosis), and unlikely (coeliac disease) infectious disease association (Mentzer et al. 2019). They observed significant seroprevalence differences among various demographics, including sex, age, lifetime sexual partners, and self-reported ethnicity (hepatitis B virus, CMV) for varying infections, and were able to confirm multiple previously reported associations with infectious diseases (Mentzer et al. 2019). Interestingly, the number of lifetime sexual partners was not associated with infectious diseases with well-established sexual routes of transmission (HIV, HBV, HCV, HTLV-1), which appears to be due to the rare occurrences of these particular diseases in this study population (Mentzer et al. 2019). However, when considering HIV and HBV in men who have sex with men, a statistically significant association was identified (Mentzer et al. 2019). The use of large, pre-established, ethnically diverse cohorts is an intriguing option for GWAS moving forward. Samples such as the UK Biobank not only have the power to detect novel variants, but can also help to address the disproportionately low number of non-European cohorts currently seen in genomics, which continues to be exacerbated through the resampling of European cohorts (Popejoy and Fullerton 2016).

Similarly, direct to consumer genomic companies, such as 23andMe also provide a potential resource for enhanced genetic discovery in multiple populations. 23andMe have currently sold more than 10 million genotyping kits, of which 80% of customers have consented to participate in ongoing research (23andMe 2019). However, their client base currently mirrors the overall ethnic diversity seen in genomic research, with the majority of their data set being Caucasian and Asian individuals (23andMe 2019; Popejoy and Fullerton 2016). To address this discrepancy, 23andMe has created multiple initiatives to increase their genetic diversity through the Global Genetics Project, Roots Into the Future, African-American Sequencing Project, and African Genetics Project (23andMe 2019). These initiatives have helped to reach a milestone of 45,000 African Americans genotyped on their platform as of May 2016 (23andMe 2019). Leveraging these data would help to increase the diversity seen in genomic research and ultimately could help to improve precision medicine options in non-Europeans. Currently, 23andMe has studied the impact of genetic variants for common infections (chickenpox, cold sores, mononucleosis, yeast infections, etc.) in a cohort of 200,000 European individuals (Tian et al. 2017). They found 13 genome-wide significant SNPs located within the HLA region, many of which were located in the peptide binding cleft, along with more than 40 SNPs located elsewhere (Tian et al. 2017). Inclusion of multiple ethnic groups in the same type of study will serve to further reduce the ancestry gap in genomic medicine.

## Precision medicine in HIV

With the expansion of available cohorts in terms of sample size, ethnic diversity, demographics, and clinical samples, precision medicine is becoming an increasingly more attainable approach. Precision medicine uses individual differences in genetics, socioeconomic factors, environment, and lifestyle to tailor health care options (disease prevention, diagnosis, treatment) to the individual, instead of the population at large which may not accurately reflect the individual. Genetic precision medicine was implemented in HIV care early on in resource-rich countries, when it was observed that individuals carrying the HLA-B*57:01 allele were at high risk for a hypersensitivity reaction to abacavir, a commonly used antiretroviral drug (Hetherington et al. 2002; Mallal et al. 2002). Pre-screening for this allele was shown to reduce this reaction in predominantly white populations (Mallal et al. 2008).

Adverse events have been observed for other commonly used antiretrovirals, including efavirenz (EFV) which was among the first drug to be co-formulated into single pill regimens for mass rollout globally. Several genetic variants in the drug-metabolizing enzyme CYP2B6 have been associated with high EFV plasma concentration and increased risked of adverse neuropsychiatric effects (Haas et al. 2004; Nyakutira et al. 2008; Rotger et al. 2005). Homozygosity, for one such variant, rs3745274, increases the risk of adverse reaction to EFV by fivefold and, given the population differences in frequency, this risk genotype is much more common in Africans (13.7%) than Europeans (5.6%) (Auton et al. 2015). Consistent with this, after the mass rollout of the new three-in-one single pill regimen containing EFV in Zimbabwe, an alarming number of patients stopping the drug due to central nervous system side effects was observed, underscoring the need to understand the distribution of such genotypes across treatment contexts (Nordling 2017). This will only become more necessary as global rollout of ART seeks to put > 80% of the 37.8 million people living with HIV on ART (Unaids 2020). Genomic pre-screening prior to ART initiation may help to reduce adverse events and could also include profiles for risk of developing long-term metabolic and cardiovascular complications known to be increased in people on ART (Rotger et al. 2010, 2013). The benefit of genomic testing prior to ART initiation will have to be weighed against concerns surrounding patient privacy and data security (Mclaren et al. 2016), however, if the diversity gap in genomics goes unaddressed, precision medicine will primarily benefit European populations. This is further underscored by a recent analysis of the performance of polygenic risk scores in predicting disease in non-European populations which showed an average 50% reduction in predictive accuracy of these scores in populations of African ancestry (Duncan et al. 2019).

One of the most ambitious initiatives in advancing precision medicine is the National Institute of Health’s All of Us Research Program. By 2024, at least 1 million diverse participants living in the United States will have contributed biospecimens, physical measurements, health and lifestyle surveys, socioeconomic data, health records, family medical history, and or wearable device data (The All of Us Research Program Investigators 2019). One of the core values of the All of Us Research Program is participant diversity and inclusiveness (The All of Us Research Program Investigators 2019). As of July 2019, more than 175,000 participants contributed biospecimens; of those participants, 51% are non-white and 80% meet the All of Us Research Program’s definition of being underrepresented in biomedical research (The All of Us Research Program Investigators 2019). Overall, this will be a historic effort in identifying new risk factors (biology, environment and social determinants) to improve diagnosis, treatment and care for diverse populations (The All of Us Research Program Investigators 2019). In addition, valuable population reference panels, such as the African Genome Project will improve ability to interpret results of genome-wide studies (Gurdasani et al. 2015). This approach should be expanded to additional valuable resources such as GTEx to better understand how genetic variability impacts gene expression in multiple cell types in diverse ethnic groups (Lonsdale et al. 2013).

## Conclusion

GWAS have been failing to accurately reflect the genetic diversity seen worldwide due to the extensive research in predominantly European populations. In recent years, there has been a concerted effort to address the gap in genomics between European and non-European cohorts, however, the discrepancy is still pronounced. Underrepresented populations have a greater risk for poorer health outcomes due to subpar prevention, diagnosis, and treatment options for their population. Through inclusivity, genomics has the opportunity to address these health disparities and improve health globally.
